# Healthcare System Drivers of Prostate Cancer Disparities: Real-World Evidence, Informatics Pathways, and Policy Translation

**DOI:** 10.3390/healthcare14182975

**Published:** 2026-09-11

**Authors:** Chen Yang, Zelin Guo, Fan Cheng, Yonghui Wan, Yan Liu

**Affiliations:** 1 Department of Urology, Renmin Hospital of Wuhan University, Wuhan 430060, China; cynthia903@yeah.net; 2 Cancer Center, Renmin Hospital of Wuhan University, Wuhan 430060, China; 3 Department of Urology, Guiqian International Hospital, Guiyang 550002, China; 2023283020246@whu.edu.cn; 4 Taikang Center for Life and Medical Sciences, Wuhan University, Wuhan 430071, China; 5 Hubei Provincial Key Laboratory for Precision Diagnosis and Treatment of Urogenital Diseases and Medical-Engineering Integration, Wuhan 430060, China; 6 Department of Nursing, Renmin Hospital of Wuhan University, Wuhan 430060, China

**Keywords:** prostate cancer, healthcare systems, health disparities, implementation science, nursing informatics, SEER–Medicare, NCDB, EHR surveillance, policy translation

## Abstract

**Background**: Racial and socioeconomic differences in prostate cancer outcomes are often described as survival disparities, but observed differences may also reflect healthcare access, treatment delivery, and resource allocation. **Methods**: NCDB and SEER–Medicare were treated as independent, complementary retrospective data sources without individual-level linkage. The analytic material comprised 111,396 database records from 2010 to 2020 across two independent source files; this is a cross-source record count, not a deduplicated count of unique persons. Harmonized descriptive analyses characterized stage, treatment, and treatment timing, while multivariable Cox proportional hazards modeling evaluated prostate cancer-specific survival (CSS) in outcome-eligible SEER–Medicare records. The CSS model denominator is therefore substantially smaller than the total record count, and the two should not be confused. **Results**: Records for non-Hispanic Black (NHB) men more often showed stage III–IV disease than records for non-Hispanic White (NHW) men (26.9% vs. 18.7%) and lower receipt of guideline-concordant first-course management initiated within 90 days, particularly in the low-SES subgroup (51.4% vs. 84.3% among high-SES NHW men). In the adjusted CSS model, NHB race was associated with higher mortality (HR = 1.32; 95% CI: 1.24–1.41). Uninsured status (HR = 1.47; 95% CI: 1.33–1.62), low income (HR = 1.28; 95% CI: 1.19–1.37), and rural residence (HR = 1.14; 95% CI: 1.06–1.22) were also associated with higher mortality. **Conclusions**: The findings support a healthcare-system interpretation of prostate cancer disparities and identify measurable targets for prospective evaluation, including EHR-enabled monitoring, nurse navigation, telehealth-supported follow-up, and facility-level treatment-concordance review.

## 1. Introduction

Prostate cancer remains a common malignancy and a major cause of cancer mortality among men in the United States. NHB men experience higher incidence and mortality than NHW men, while low-income, uninsured, and rural patients face additional barriers across the cancer-care continuum [1,2,3,4,5,6]. The broader health-equity literature links socioeconomic position, insurance, and barriers to care with unequal health outcomes [7,8,9,10,11,12]. Prostate-specific observational studies have documented racial and socioeconomic differences in disease characteristics, treatment, and survival [13,14,15,16,17,18,19,20,21,22,23]. The present study adds a source-aware health-system analysis rather than another undifferentiated pooled disparity estimate.

Prostate cancer disparities may reflect both sociobiological factors [13,14,15] and healthcare-system or access factors [16,17,18,19,20,21]. Equal-access and clinical-trial cohorts show that racial outcome gaps can narrow when access and treatment are more comparable [17,18,22], and systematic review evidence supports a multifactorial interpretation [23]. Differences in early detection also shape the stage at which prostate cancer is identified, reflecting guideline-based early-detection recommendations, shared decision making for PSA testing, screening-trial evidence, pretreatment risk assessment, and the diagnostic follow-up of high-grade precursor findings [24,25,26,27,28]. The unresolved question is which measurable access and delivery patterns remain observable across real-world data sources with different populations, treatment information, and survival endpoints.

The two databases were used because they contribute different information rather than because they form one pooled cohort. Compared with a single-database NCDB or SEER analysis, this design uses NCDB for broad facility-based diagnosis and first-course treatment information and SEER–Medicare for population-based registry/claims information and cause-specific survival. Concordant patterns across the sources are treated as triangulation, while source-specific outcomes remain source-specific. The prespecified hypothesis was that poorer socioeconomic access and treatment delivery would be associated with later-stage presentation, lower receipt of risk-appropriate first-course management, longer treatment initiation, and poorer CSS.

The primary confirmatory objective was to estimate adjusted associations of race/ethnicity and socioeconomic indicators with CSS in outcome-eligible SEER–Medicare records. Secondary descriptive objectives were to characterize the stage at diagnosis, treatment modality, guideline-concordant first-course management, and time to treatment across the harmonized source files. Policy translation was exploratory and was used to generate implementation hypotheses for future prospective testing; it was not treated as evidence that the proposed interventions were effective in the present retrospective data.

## 2. Methods

### 2.1. Data Sources and Cohort Handling

The study used two independent and complementary data sources and did not perform patient-level linkage or cross-source deduplication. NCDB contains facility-based records from cancer programs accredited by the Commission on Cancer, whereas SEER–Medicare links population-based cancer registry information to Medicare claims for eligible beneficiaries. Each source was processed in a separate analytic file. Harmonization standardized variable definitions for comparison and descriptive synthesis; it did not create a single patient-level cohort. The reported total of 111,396 is therefore the arithmetic count of records across the two source files, not a count of 111,396 unique persons.

Eligible records represented men diagnosed with primary prostate adenocarcinoma (ICD-O-3 C61.9) from 1 January 2010 through 31 December 2020 (Table 1). NCDB eligibility included men aged 40 years or older; SEER–Medicare followed its Medicare beneficiary eligibility structure. Within each source, records with a prior malignancy, synchronous cancer, implausible stage or treatment data, missing primary outcome information, or missing required race/ethnicity, income, insurance, or treatment fields were excluded. Source-specific fields were mapped to common categories for race/ethnicity, area-level SES, insurance, rurality, clinical stage, Gleason score, PSA, treatment modality, and treatment timing. Before cross-source interpretation, the databases were compared conceptually on age eligibility, insurance capture, facility ascertainment, treatment coding, follow-up structure, and outcome availability. Because these structural differences are intrinsic to the databases, cross-source agreement was interpreted as triangulation rather than as evidence from a pooled or deduplicated cohort.

**Figure 1:**
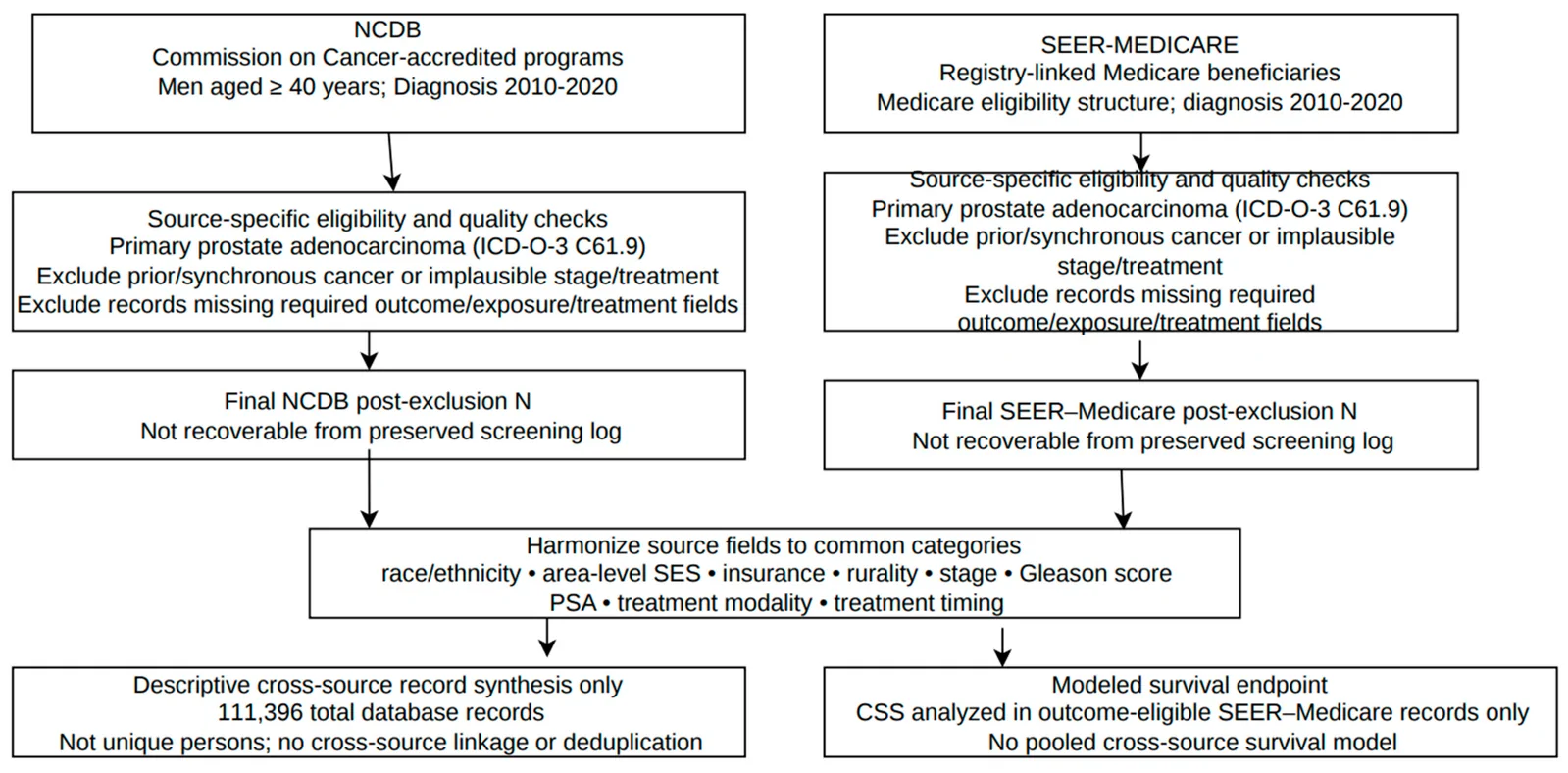
Participant-selection and source-aware cohort-handling workflow. NCDB and SEER–Medicare were retained as independent analytic files. The total of 111,396 represents cross-source database records, not unique persons. Source-specific final denominators were not recoverable from the preserved screening log; no cross-source individual linkage or deduplication was performed. CSS modeling was restricted to outcome-eligible SEER–Medicare records.

NCDB and SEER–Medicare are independent data sources with different sampling frames. NCDB includes cases from Commission on Cancer (CoC)-accredited facilities nationwide, whereas SEER–Medicare covers population-based registry data from selected geographic areas (approximately 35% of the US population) linked to Medicare claims. Although both databases contain prostate cancer cases, their catchment areas are not identical, and individual-level patient identifiers are not available to the authors for cross-source linkage. We therefore cannot perform deterministic or probabilistic deduplication. The databases were retained as separate analytic files and were not concatenated into a single patient-level cohort. Descriptive comparisons across the two sources are used for triangulation, i.e., to assess whether similar patterns of stage and treatment are observable in both data environments, rather than to create a pooled patient cohort. The CSS Cox model was restricted exclusively to SEER–Medicare records because NCDB does not contain cause-specific death information; its vital-status field records only overall survival (dead/alive) and does not distinguish cancer-specific from other-cause mortality. Because the CSS model uses only SEER–Medicare records, potential overlap with NCDB does not affect the survival estimates. Because the two sources serve different analytical roles (NCDB for broad facility-based treatment description; SEER–Medicare for cause-specific survival), potential duplication between sources does not materially affect the interpretation of the study findings. Table 1 summarizes the role of each database, the provenance of each reported outcome, and the harmonization rules applied across the two sources, and Figure 1 shows the participant-selection and source-aware cohort-handling workflow.

### 2.2. Exposure Variables

The principal exposures were race/ethnicity and socioeconomic indicators. Race/ethnicity was represented by the categories available in the source data. Socioeconomic access was represented by census-tract median household income, insurance status, educational attainment, and federal poverty level; these variables were retained as separate observed covariates to preserve clinical and policy interpretability. Additional covariates included age, PSA level, Gleason score, clinical stage, Charlson Comorbidity Index, rural–urban classification, facility type, marital status, and year of diagnosis.

The SES and access variables also correspond to fields that may be incorporated into future EHR-enabled disparity surveillance, including insurance type, area-level income, race/ethnicity, rurality, and treatment timing. No AI or ML prediction model was developed, deployed, or locally validated in this retrospective study. Any future predictive implementation would require independent local validation, subgroup calibration, bias auditing, and ongoing performance monitoring before clinical use.

### 2.3. Outcome Variables

The primary modeled outcome was CSS. Secondary descriptive outcomes were treatment modality, time to treatment initiation, and receipt of guideline-concordant first-course management initiated within 90 days of diagnosis. For the harmonized database measure, treatment concordance was classified using risk-treatment principles consistent with the AUA/ASTRO/SUO guideline for clinically localized prostate cancer [29] and the APCCC consensus for advanced disease [30]. Localized low-risk disease was considered concordant when active surveillance/watchful waiting or an appropriate definitive local therapy was recorded; intermediate- or high-risk disease was considered concordant when prostatectomy or radiation-based treatment was recorded, with ADT when clinically indicated by the available risk fields; and metastatic disease was considered concordant when a systemic-therapy pathway was recorded. The 90-day interval was an analytic timeliness threshold applied consistently across sources, not a guideline-mandated treatment deadline. Because registry and claims fields do not capture every clinical factor used in guideline decision making, this variable should be interpreted as a pragmatic secondary-data proxy for risk-appropriate first-course management rather than a chart-level guideline compliance audit. Treatment and treatment-timing variables were available in both sources after harmonization; CSS was analyzed from SEER–Medicare cause-specific outcome information. For the multivariable CSS model, treatment was specified as a categorical variable with five mutually exclusive categories based on the primary definitive first-course treatment recorded in SEER–Medicare: (1) radical prostatectomy (reference), (2) EBRT without ADT, (3) ADT alone without definitive local therapy, (4) no definitive treatment recorded, and (5) other/combined definitive therapy (including brachytherapy, combined EBRT + brachytherapy, and combined surgery + adjuvant therapy). Active surveillance/watchful waiting was included in the ‘no definitive treatment’ category for the purpose of the survival model because it does not represent definitive curative intervention. Treatment selection in localized disease is also shaped by comorbidity and life expectancy, comparative-effectiveness considerations, and patient preference [31,32,33]. Chemotherapy without ADT or local therapy was grouped with ‘other/combined’ when it was the primary recorded first-course modality; when chemotherapy accompanied ADT, it remained in the ADT-alone category because the registry treatment fields do not reliably distinguish chemotherapy with versus without ADT as primary systemic therapy. This categorization was chosen to preserve clinically meaningful distinctions while maintaining adequate cell sizes for stable estimation. The small number of records receiving brachytherapy as the sole definitive modality were included in the ‘other/combined’ category. All treatment variables in the model are mutually exclusive.

### 2.4. Statistical Analysis

Descriptive statistics characterized race/ethnicity, SES, stage, treatment, and treatment-timing patterns in the harmonized record-level summaries. These summaries were used for cross-source triangulation and were not treated as a deduplicated patient-level pooled analysis. The reported CSS model used Cox proportional hazards regression in outcome-eligible SEER–Medicare records to estimate adjusted associations with race/ethnicity, SES indicators, treatment, and clinical covariates. The model adjusted for age, Gleason score, clinical stage, PSA, comorbidity, facility type, year of diagnosis, and the listed socioeconomic and treatment covariates; robust variance estimation was used where facility clustering was applicable. Hazard ratios are interpreted as adjusted associations, not causal treatment effects.

Only analyses supported by verifiable coefficient-level output are reported. Structural equation modeling, mediation analysis, population attributable fraction analysis, and pooled cross-database survival modeling are not included because the available analytic output did not contain the coefficient-level, counterfactual, and diagnostic information required for reproducible reporting. Inference is therefore limited to the descriptive summaries and the verifiable multivariable CSS estimates shown in the manuscript.

The reported analyses use complete-case records for the variables required by each model. Records missing a required variable for a reported analysis were excluded according to the eligibility rules above. No multiple-imputation estimates are retained, and no imputation convergence results or imputed-versus-complete-case comparisons are presented. Analyses were performed using R 4.3.1 and SAS 9.4, with two-sided tests and α = 0.05.

### 2.5. Missing-Data and Sensitivity-Analysis Reporting

The preserved analytic output does not contain variable-level pre-exclusion missingness percentages, a reproducible multiple-imputation specification, convergence traces, or quantitative sensitivity-analysis outputs. These quantities were therefore neither reconstructed nor inferred. Table 2 states explicitly which missing-data and sensitivity-analysis information is available for the reported analyses and which information is unavailable.

## 3. Results

### 3.1. Cohort Characteristics

Throughout Section 3, descriptive comparisons of stage, treatment, and patient characteristics are based on the combined record-level material (111,396 records across both sources) for triangulation. All survival estimates and Cox model results are based exclusively on the outcome-eligible SEER–Medicare subset, as specified in Section 2.3.

The analytic material comprised 111,396 database records from 2010 to 2020 across the two independent source files. This total is a record inventory and should not be interpreted as 111,396 unique persons because cross-source linkage and deduplication were not performed. The combined descriptive record distribution was NHW 52.3% (*n* = 58,241), NHB 17.8% (*n* = 19,847), Hispanic 12.7% (*n* = 14,203), API 6.8% (*n* = 7577), and Other/Unknown 10.4% (*n* = 11,528). Table 3 presents the three largest racial/ethnic groups as a descriptive harmonized record-level summary; inferential survival estimates are source-specific as described in Table 1.

NHB men were younger at diagnosis (mean 63.8 vs. 67.2 years; *p* < 0.001), more often lived in low-income areas (38.6% vs. 18.4%; *p* < 0.001), were more often uninsured (8.3% vs. 4.1%; *p* < 0.001), and more often presented with high-grade disease (Gleason ≥8: 31.4% vs. 22.1%; *p* < 0.001). Stage III–IV disease was observed in 26.9% of NHB men versus 18.7% of NHW men (*p* < 0.001). Hispanic men generally showed intermediate clinical patterns but had the highest uninsured proportion (14.7%).

### 3.2. Treatment Patterns by Race and Socioeconomic Status

Table 4 summarizes harmonized, descriptive treatment-delivery patterns across the independent source files. Radical prostatectomy was recorded for 38.4% of the NHW records, 28.1% of the NHB records, and 31.6% of Hispanic records (*p* < 0.001). Active surveillance/watchful waiting was less frequently recorded for NHB than NHW records; this difference should be interpreted in the context of the higher frequency of high-risk presentation and the pragmatic, risk-adapted definition of concordance.

NHB records more often had no definitive first-course treatment recorded (9.3% vs. 4.7%; *p* < 0.001) and had a longer mean interval to treatment initiation (58.7 ± 22.4 vs. 42.3 ± 18.6 days; *p* < 0.001). Academic-center treatment was less frequent in NHB than NHW records (28.7% vs. 41.3%; *p* < 0.001). The lowest recorded proportion of guideline-concordant first-course management initiated within 90 days was observed among low-SES NHB records (51.4%) compared with high-SES NHW records (84.3%). Uninsured records also showed longer treatment intervals and lower surgical treatment rates. These are descriptive access patterns and do not establish a causal effect of insurance or race on treatment selection.

### 3.3. Prostate Cancer-Specific Survival by Race/Ethnicity and SES

**Figure 2:**
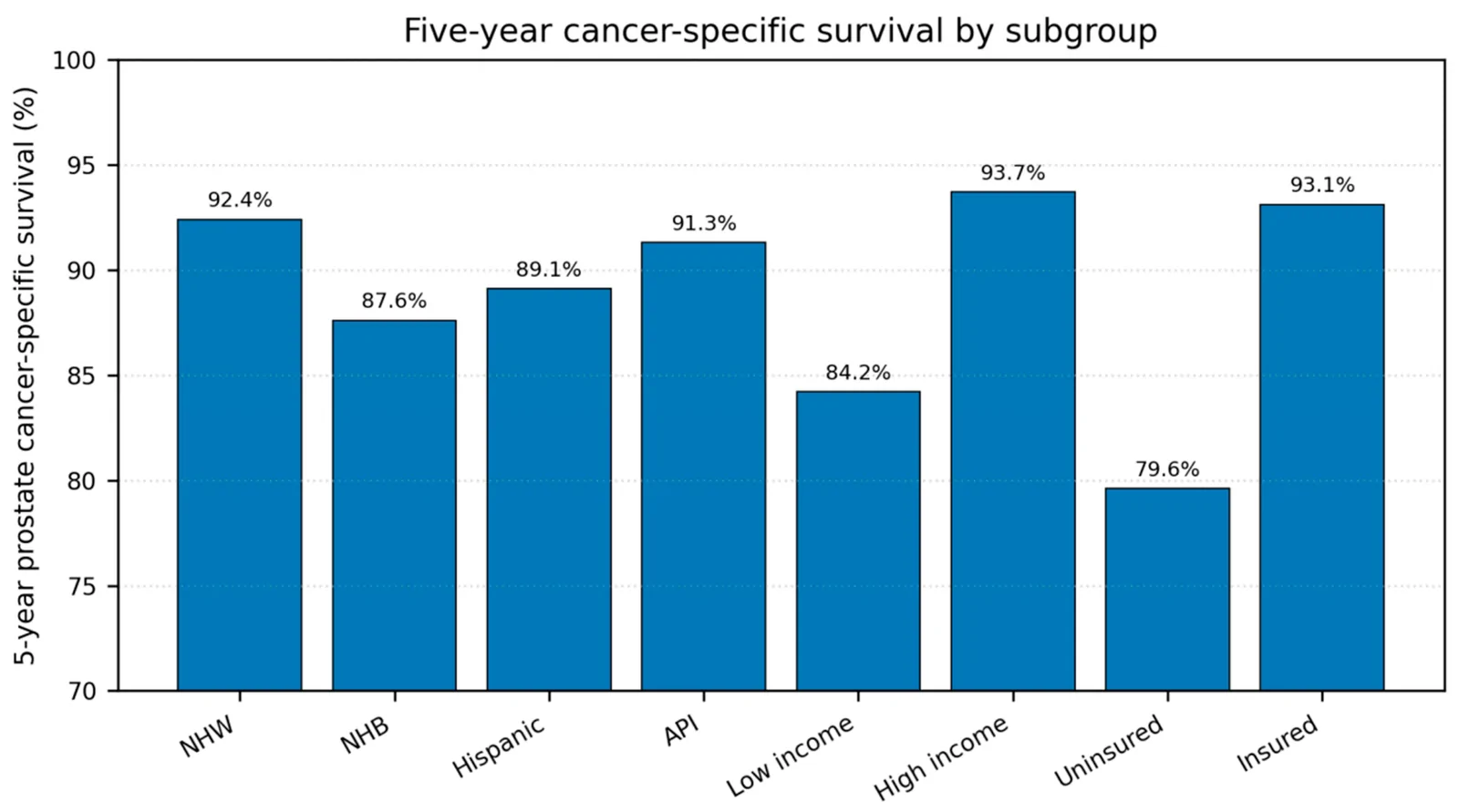
Five-year prostate cancer-specific survival (%) by race/ethnicity and selected SES indicators. These survival estimates are derived exclusively from outcome-eligible SEER–Medicare records, not from the combined 111,396 record-level descriptive material. Unadjusted estimates from outcome-eligible SEER–Medicare records; bars show point estimates. Confidence intervals were produced in the underlying analysis but are not reproduced in the figure.

**Figure 3:**
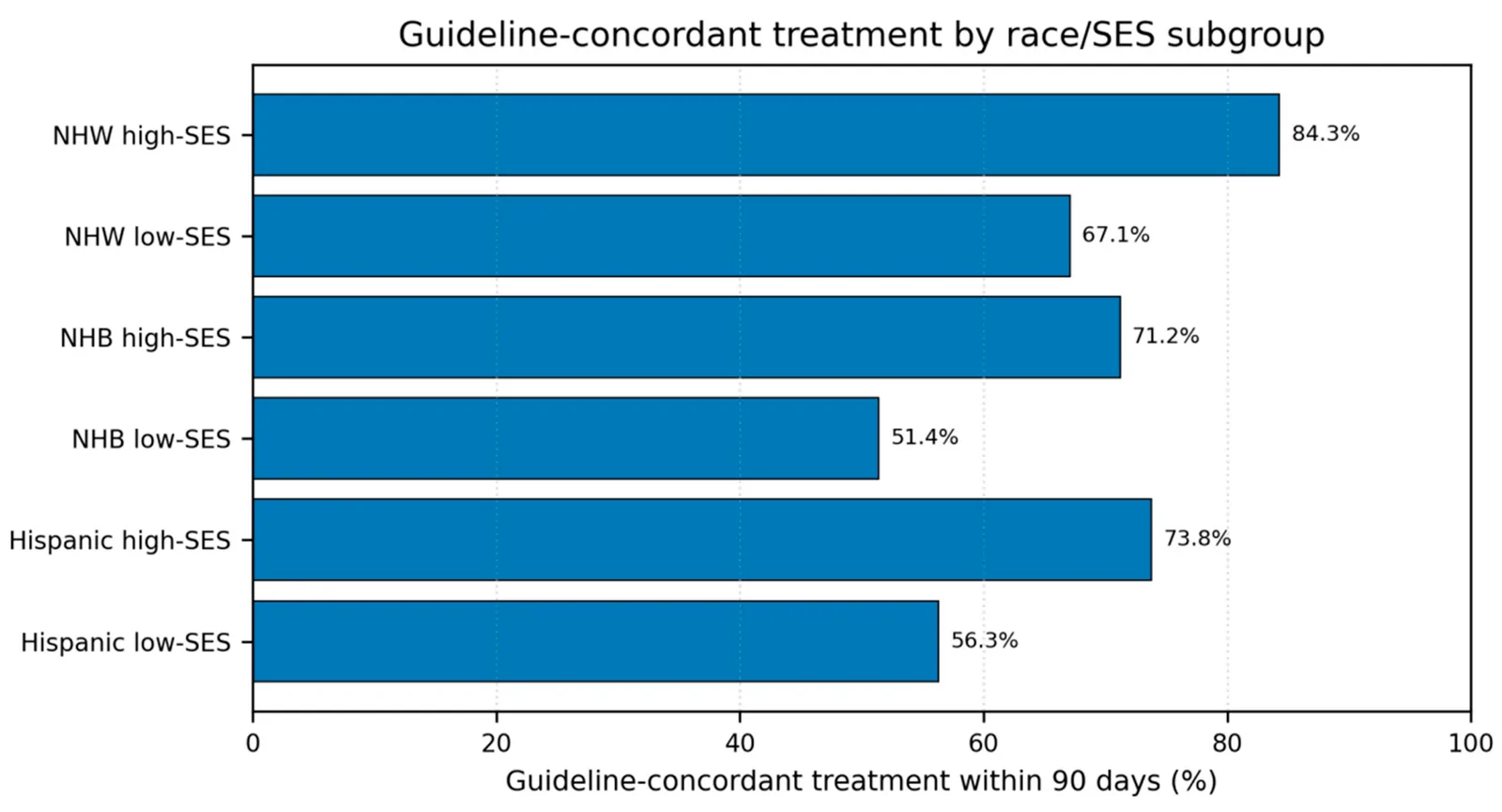
Percentage receiving risk-appropriate first-course prostate cancer management initiated within 90 days of diagnosis, stratified by race/ethnicity and SES. This is a descriptive harmonized record-level measure using AUA/ASTRO/SUO-consistent localized-disease categories [29] and APCCC-consistent advanced-disease categories [30]. The 90-day window is an analytic timeliness threshold, not a guideline-mandated deadline. NHW = non-Hispanic White; NHB = non-Hispanic Black.

**Figure 4:**
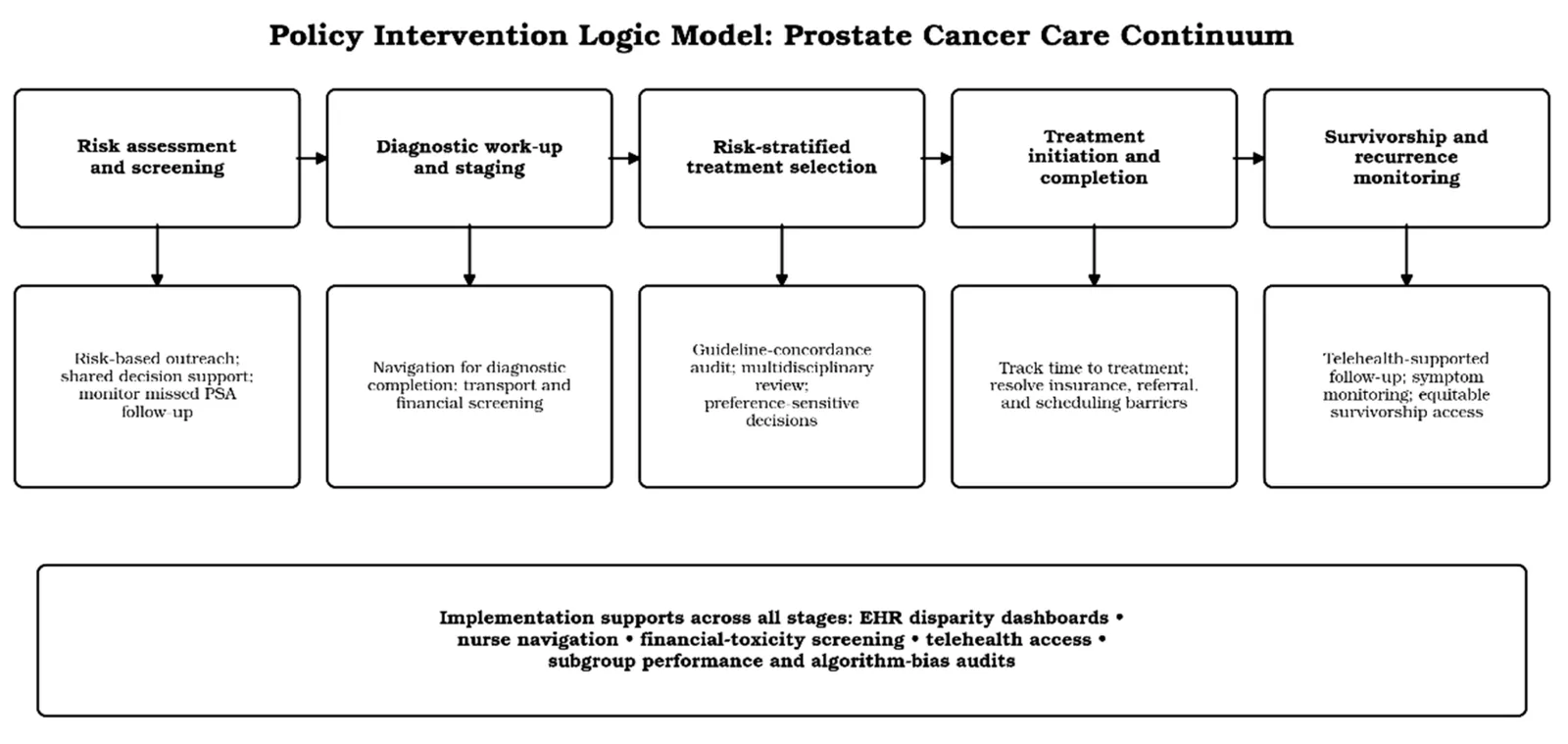
Policy intervention logic model for the prostate cancer-care continuum. The round bullet symbols (•) in the lower panel are list separators between the implementation supports and carry no additional meaning; they do not denote sequence, weighting, or a separate model component.

Figure 2 presents unadjusted five-year CSS from outcome-eligible SEER–Medicare records. The five-year CSS was lower for NHB records (87.6%) than for NHW (92.4%), Hispanic (89.1%), and API (91.3%) records. Differences were also observed by insurance status (79.6% uninsured vs. 93.1% insured) and income (84.2% low-income vs. 93.7% high-income). These estimates are descriptive and precede multivariable adjustment. Figure 3 presents the corresponding proportions of records with risk-appropriate first-course management initiated within 90 days of diagnosis, and Figure 4 summarizes the policy intervention logic model developed from the observed access patterns.

The logic model maps risk assessment, diagnostic work-up, treatment selection, treatment initiation, and survivorship to potential implementation supports, including EHR monitoring, navigation, financial screening, telehealth, and equity audits. It is an implementation-planning framework derived from the observed access patterns and prior literature, not an empirical result of the database analyses.

### 3.4. Multivariable Analysis: Independent Predictors of Survival

Table 5 presents the adjusted Cox model for CSS in outcome-eligible SEER–Medicare records. NHB race was associated with a higher prostate cancer-specific mortality hazard after adjustment for age, Gleason score, clinical stage, PSA, comorbidity, year of diagnosis, facility type, socioeconomic indicators, and treatment variables (HR = 1.32; 95% CI: 1.24–1.41; *p* < 0.001). Hispanic and API race/ethnicity were associated with lower adjusted hazards relative to NHW records in this model.

Socioeconomic and delivery-related variables were also associated with CSS. Uninsured status (HR = 1.47; 95% CI: 1.33–1.62), low income (HR = 1.28; 95% CI: 1.19–1.37), and rural residence (HR = 1.14; 95% CI: 1.06–1.22) were associated with higher mortality hazards. Advanced stage and high Gleason score showed the largest clinical associations. The ‘No Treatment’ category (HR = 2.87; 95% CI: 2.61–3.15) must not be interpreted as the causal effect of withholding beneficial therapy. It may include patients with extensive metastatic disease, severe comorbidity, limited life expectancy, contraindications, treatment refusal, or preference-sensitive goals of care for whom definitive treatment was inappropriate. Confounding by indication, competing mortality risk, and residual disease-severity differences are therefore likely.

## 4. Discussion

The observed patterns support a healthcare-system interpretation of prostate cancer disparities while remaining consistent with a multifactorial model of risk. The stage at presentation, treatment receipt, treatment timing, facility access, and CSS varied with insurance, income, rurality, and facility characteristics. Because the study is retrospective and source-specific data structures differ, these findings identify associations and implementation hypotheses rather than causal mechanisms.

### 4.1. Key Findings

The records for NHB and lower-SES men more often showed later-stage disease, longer treatment-initiation intervals, lower receipt of risk-appropriate first-course management, and poorer CSS. These patterns are consistent with prior prostate cancer studies of nondefinitive therapy, treatment access, pelvic lymph-node assessment, and facility-associated disparities [34,35,36,37,38,39]. The contribution of the present study is source-aware triangulation: it separates descriptive cross-source patterns from the source-specific survival model and from the subsequent implementation proposals.

### 4.2. Plausible Mechanisms

Several mechanisms may plausibly contribute to these associations. Insurance status can shape access to diagnostic evaluation and treatment; facility type and location can determine specialist and technology availability; and financial toxicity can delay or interrupt care. Tumor biology, disease severity, comorbidity, and patient preferences also remain important. Advanced prostate cancer increasingly requires multidisciplinary, resource-intensive care and access to contemporary systemic therapy [30,40]. These mechanisms are plausible explanations to be tested prospectively, not mediating effects established by the present data.

The large hazard ratio associated with the ‘No Treatment’ category requires particular caution. This variable records the absence of definitive first-course treatment, not the absence of clinical care. Patients may have been managed palliatively, may have had disease too advanced for definitive therapy, may have had severe comorbid illness or limited life expectancy, or may have declined treatment after shared decision making. Treatment eligibility and the goals of care are incompletely captured in the databases. The HR of 2.87 should therefore be interpreted primarily as a marker of case mix and confounding by indication rather than as evidence that a health system failed to treat every patient who could benefit.

### 4.3. Health Informatics and AI-Driven Disparity Surveillance

The measured variables could support future EHR-enabled disparity surveillance. Health systems could use rule-based dashboards to monitor risk-appropriate first-course management, treatment initiation, missed PSA follow-up, navigation referrals, and financial-toxicity screening across patient groups. Clinical decision support could then prompt referral to oncology navigation, financial counseling, transportation assistance, or telehealth services when predefined care gaps are detected. These proposals require prospective evaluation and were not tested by the present database analysis.

AI/ML risk stratification should be considered only after reliable SDOH data capture, local validation, and continuous subgroup performance monitoring are established. Race should not be treated as a biological risk proxy; its inclusion can encode structural inequities and produce unequal error rates. Governance should therefore include transparent feature review, subgroup calibration, fairness audits, documentation of false-positive and false-negative rates, and human oversight. No predictive model was developed, deployed, or locally validated in this study.

### 4.4. Nursing-Led Interventions Across the Care Continuum

Nursing-led navigation is a plausible delivery strategy for the access barriers identified in the study. Oncology nurse navigators can assess transportation, insurance, language, financial, and scheduling barriers; coordinate referrals and treatment preparation; support shared decision making; and follow patients through treatment and survivorship. EHR-based referral rules could prioritize patients with documented access barriers, but the effectiveness and equity impact of such workflows require prospective testing.

### 4.5. Telehealth and Digital Technology

Telehealth may reduce selected follow-up barriers for rural or transportation-limited patients by supporting remote PSA review, urology consultation, medication monitoring, and survivorship care. Its benefit depends on broadband access, devices, language services, digital support, reimbursement, and the availability of in-person pathways when examination, biopsy, imaging, or treatment is required.

Evaluation should therefore include both access and failure metrics, such as completed visits, failed connections, broadband-related failures, PSA-test completion, conversion to in-person care, and subgroup differences in follow-up completion. Telehealth should be treated as a supplement to, not a replacement for, necessary in-person oncology care.

### 4.6. Implementation Science Framework

RE-AIM and CFIR provide complementary frameworks for prospective evaluation of system-level interventions. RE-AIM can assess reach, effectiveness, adoption, implementation, and maintenance [41], whereas CFIR can assess organizational readiness, leadership engagement, available resources, workflow compatibility, and patient needs [42]. These frameworks are proposed for future implementation studies because the present retrospective analysis did not estimate intervention effectiveness.

A phased implementation strategy would begin with lower-complexity, auditable workflows such as standardized referral criteria, nurse navigation, and rule-based disparity dashboards. Telehealth and financial-navigation workflows could then be added with predefined reach and equity metrics. Predictive AI should be considered only after the underlying data, governance, and validation processes have demonstrated acceptable performance in relevant subgroups.

Although the empirical claims in this manuscript remain prostate-cancer-specific, the implementation challenges are shared across complex healthcare settings. Policy work in pediatric congenital heart disease emphasizes standardized management protocols and multidisciplinary oversight for medication-related risks [43]. Health-policy work on ML similarly highlights standardized data, rigorous validation, interpretability, fairness oversight, and regulatory alignment [44]. In cancer supportive care, technology implementation is also constrained by reimbursement, workforce training, access, and governance [45]. These cross-domain parallels support the implementation logic of phased deployment, multidisciplinary governance, validation, and equity monitoring; they are not used as evidence for prostate-cancer effect estimates.

### 4.7. Policy and Implementation Implications

The policy relevance of the study is therefore limited to translating observed associations into testable delivery-system strategies. Insurance, treatment timing, and facility-access signals arise directly from the present data; patient navigation and financial-burden assessment are supported by the prior cancer-care literature [46,47]; and EHR dashboards, telehealth workflows, and AI governance are evidence-informed implementation proposals. Table 6 labels these evidence sources explicitly so that proposed interventions are not presented as effects demonstrated by the current study.

### 4.8. Limitations

Several limitations should guide interpretation. First, the retrospective observational design does not establish causality. Second, NCDB and SEER–Medicare have different sampling frames, age structures, insurance information, facility representation, and outcome availability; no individual-level linkage or cross-source deduplication was possible. Third, the preserved source-screening output did not include final database-specific denominators or variable-level pre-exclusion missingness percentages, which limits assessment of selection across sources. Fourth, reproducible multiple-imputation specifications, convergence diagnostics, and quantitative sensitivity outputs were unavailable. Fifth, area-level SES measures may not capture individual financial circumstances, transportation, digital access, or social support. Race/ethnicity categories do not represent biological ancestry and may reflect unmeasured social and healthcare-system exposures. Organizational culture, local service capacity, staffing, referral-network performance, clinician communication, treatment eligibility, and patient preferences were incompletely measured. Finally, changes in prostate cancer guidance and telehealth policy over 2010–2020 may limit direct extrapolation to current practice.

## 5. Policy Recommendations

Table 6 summarizes six implementation options and explicitly distinguishes present-study associations from prior cancer-care evidence and expert implementation interpretation. Each option is paired with foreseeable barriers, feasibility considerations, sustainability indicators, and EHR-based monitoring measures. None of the proposed interventions was evaluated for effectiveness in the present retrospective study.

These recommendations are quality-improvement hypotheses for prospective evaluation. Future studies should prespecify equity, reach, effectiveness, adoption, fidelity, safety, and maintenance outcomes and should report results by relevant patient subgroups.

## 6. Conclusions

This study positions prostate cancer disparities as a healthcare-system and policy issue while preserving the limits of retrospective secondary data. Across complementary real-world data sources, records for NHB, lower-income, uninsured, and rural men showed less favorable stage, treatment, treatment-timing, facility-access, and CSS patterns. The adjusted SEER–Medicare CSS model also showed associations with race/ethnicity and socioeconomic indicators. These findings do not establish that a single system factor caused the survival differences. They identify measurable targets for prospective evaluation, including EHR-based disparity surveillance, nurse navigation, financial-toxicity screening, telehealth-supported follow-up, and facility-level treatment-concordance review. Future studies should validate these strategies prospectively using RE-AIM and CFIR measures and should report subgroup-specific reach, effectiveness, adoption, fidelity, and maintenance.

## Figures and Tables

**Table 1:** Database roles, outcome provenance, and source-aware harmonization strategy.

Domain	NCDB Role	SEER–Medicare Role	Harmonization/Analysis Rule
Population/eligibility	Facility-based cancer records; men aged ≥40 years; 2010–2020	Registry-linked Medicare beneficiaries; Medicare eligibility structure; 2010–2020	Sources retained as independent analytic files; age structures were not forced to match.
Source-specific final denominator	Not recoverable from the preserved source-screening log	Not recoverable from the preserved source-screening log	111,396 is the arithmetic total of source records, not unique persons; no cross-source linkage or deduplication.
Stage and tumor features	Available	Available	Mapped to common clinical stage, Gleason score, and PSA categories for descriptive comparison.
Treatment and time to treatment	Available	Available from registry/claims fields	Mapped to common modality groups and days from diagnosis to first treatment; cross-source summaries are descriptive.
Guideline-concordant first-course management	Derived	Derived	Risk-treatment categories consistent with AUA/ASTRO/SUO localized guidance [29] and APCCC advanced-disease consensus [30]; 90 days is an analytic timeliness threshold, not a guideline mandate.
Prostate cancer-specific survival	Not used for the CSS model	Modeled survival source	Cox modeling restricted to outcome-eligible SEER–Medicare records; no pooled cross-source survival model.
Overall survival	Available in the source	Available in the source	Not analyzed as an inferential endpoint in this report because source-specific plotted/model outputs were not available for verification.

**Table 2:** Availability of missing-data and sensitivity-analysis information for the reported analyses.

Status/Rationale	Reporting Approach	Item
Verifiable from the preserved manuscript output.	Complete-case records for the variables required by each reported analysis	Analysis population
Percentages were not present in the preserved source-level screening output and were not estimated.	Not reported	Variable-level pre-exclusion missingness
A reproducible imputation specification was unavailable; therefore, multiple imputation was not included.	Not used; number of imputed datasets = 0	Multiple imputation
No imputed analysis is retained.	Not applicable	Imputation variables and convergence
No imputed analysis is retained.	Not applicable	Imputed-versus-complete-case comparison
Numerical sensitivity-analysis outputs were unavailable; sensitivity analyses are therefore not reported.	Not reported	Quantitative sensitivity analyses

**Table 3:** Descriptive baseline characteristics by race/ethnicity for the three largest groups (*N* = 92,291 records). Counts are harmonized record-level summaries across the independent source files and are not cross-source deduplicated.

Characteristic	Non-Hispanic White	Non-Hispanic Black	Hispanic	*p*-Value
Total Patients, *n* (%)	58,241 (52.3%)	19,847 (17.8%)	14,203 (12.7%)	<0.001
Mean Age at Diagnosis (years)	67.2 ± 9.4	63.8 ± 9.1	65.4 ± 9.8	<0.001
Low-Income (<200% FPL), %	18.4%	38.6%	41.2%	<0.001
No Insurance, %	4.1%	8.3%	14.7%	<0.001
Urban Residence, %	71.3%	84.2%	88.6%	<0.001
Gleason Score ≥ 8 at Diagnosis, %	22.1%	31.4%	24.8%	<0.001
Stage III–IV at Diagnosis, %	18.7%	26.9%	23.1%	<0.001
PSA > 20 ng/mL at Diagnosis, %	14.3%	22.8%	17.9%	<0.001
Any Comorbidity (CCI ≥ 2), %	34.6%	41.2%	38.7%	<0.001
Married/Partnered, %	72.8%	46.3%	63.5%	<0.001

Note: Abbreviations: FPL = federal poverty level; CCI = Charlson Comorbidity Index; PSA = prostate-specific antigen. *p*-values are record-level descriptive comparisons from chi-square tests or ANOVA, as appropriate, and should not be interpreted as evidence from a deduplicated pooled cohort. Table 3 presents descriptive record-level characteristics from both sources combined. These descriptive data are not the same sample as the CSS Cox model, which was restricted to SEER–Medicare records only.

**Table 4:** Descriptive treatment patterns by race/ethnicity and selected treatment-access indicators across harmonized source records.

Treatment Type	NHW (%)	NHB (%)	Hispanic (%)	*p*-Value
Radical Prostatectomy	38.4%	28.1%	31.6%	<0.001
Radiation Therapy (EBRT)	29.2%	33.8%	30.1%	<0.001
Brachytherapy	8.3%	4.9%	6.2%	<0.001
Active Surveillance/Watchful Waiting	14.6%	9.8%	12.4%	<0.001
Androgen Deprivation Therapy (ADT)	18.7%	24.3%	21.9%	<0.001
Chemotherapy	5.1%	7.9%	6.4%	0.003
No Treatment (deferred/refused)	4.7%	9.3%	8.1%	<0.001
Time to Treatment Initiation (days)	42.3 ± 18.6	58.7 ± 22.4	52.1 ± 20.8	<0.001
Treated at Academic Center, %	41.3%	28.7%	30.2%	<0.001

Note: Abbreviations: NHW = non-Hispanic White; NHB = non-Hispanic Black; EBRT = external beam radiation therapy; ADT = androgen deprivation therapy. *p*-values are record-level descriptive comparisons from chi-square tests for proportions and ANOVA for continuous variables. Treatment categories in Table 4 are not mutually exclusive. Patients may receive combination therapies (e.g., EBRT with ADT, or surgery with adjuvant therapy), and the percentages therefore sum to more than 100%. The table describes the proportion of records in which each treatment modality was recorded as part of first-course management; it does not represent mutually exclusive treatment assignment.

**Table 5:** Multivariable Cox proportional hazards model for prostate cancer-specific survival in outcome-eligible SEER–Medicare records. NCDB records were not included in this model because NCDB does not capture cause-specific mortality.

Variable	HR	95% CI	*p*-Value	Interpretation
Race/Ethnicity (Ref: NHW)				
non-Hispanic Black	1.32	1.24–1.41	<0.001	Higher adjusted hazard
Hispanic	0.91	0.84–0.98	0.014	Lower adjusted hazard
Asian/Pacific Islander	0.84	0.76–0.93	<0.001	Lower adjusted hazard
Socioeconomic Factors				
Low Income (<200% FPL)	1.28	1.19–1.37	<0.001	Higher adjusted hazard
No Insurance	1.47	1.33–1.62	<0.001	Higher adjusted hazard
Rural Residence	1.14	1.06–1.22	0.001	Higher adjusted hazard
Clinical Factors				
Gleason Score ≥ 8	2.41	2.28–2.54	<0.001	Higher adjusted hazard
Stage III–IV	3.18	2.97–3.40	<0.001	Higher adjusted hazard
CCI ≥ 2	1.63	1.54–1.73	<0.001	Higher adjusted hazard
Treatment (Ref: Radical Prostatectomy)				
No Treatment	2.87	2.61–3.15	<0.001	Strongly elevated association
ADT Only	1.74	1.63–1.85	<0.001	Elevated association
EBRT	1.12	1.04–1.20	0.002	Slightly elevated association

Note: Abbreviations: HR = hazard ratio; CI = confidence interval; NHW = non-Hispanic White (reference); FPL = federal poverty level; CCI = Charlson Comorbidity Index; ADT = androgen deprivation therapy; EBRT = external beam radiation therapy. Model adjusted for age, Gleason score, clinical stage, PSA, facility type, year of diagnosis, and all listed covariates simultaneously. Associations should not be interpreted as causal treatment effects. Treatment categories in the Cox model are mutually exclusive and based on the primary definitive first-course treatment recorded in SEER–Medicare. Radical prostatectomy is the reference category. “ADT Only” includes ADT without definitive local therapy. “EBRT” includes EBRT without ADT. “No Treatment” includes active surveillance/watchful waiting and records with no definitive treatment documented. “Other/combined” (not shown in the table due to small sample size and non-significant association) includes brachytherapy alone, combined EBRT + brachytherapy, and combined surgery + adjuvant therapy. Complete category definitions are provided in Section 2.3.

**Table 6:** Streamlined implementation recommendations for healthcare systems and policy translation.

Recommendation	Evidence Basis	Implementation Barriers	Feasibility Considerations	Sustainability Indicators	EHR Monitoring Metrics
EHR-enabled disparity surveillance and clinical decision support	Present study: treatment and survival patterns differed by insurance, SES, rurality, facility, and race/ethnicity. Expert interpretation: dashboards can convert these signals into measurable care gaps.	Incomplete SDOH data; inconsistent race/ethnicity coding; limited informatics staffing; risk of algorithmic bias and differential error rates.	Begin with rule-based dashboards; establish data-quality thresholds; require subgroup calibration, fairness audits, and human oversight before predictive AI.	Quarterly dashboard review; data completeness; documented action plans; subgroup performance audits.	Treatment initiation within 90 days; TTI; missed PSA follow-up; referral completion; metrics stratified by race/ethnicity, insurance, SES, rurality, and facility.
Nurse navigation for high-risk access groups	Present study: lower treatment concordance and longer TTI among NHB and lower-SES records. Prior literature supports navigation for appointment, insurance, and logistical barriers [46].	Navigator staffing; reimbursement uncertainty; role overlap; high caseloads.	Use transparent EHR referral criteria for uninsured, low-income, rural, or clinically high-risk patients; integrate social work and financial counseling.	Navigator caseload; contact within 7 days; referral closure; treatment initiation within 90 days.	Navigation referral date; first contact date; documented barriers; treatment start date; care-plan completion.
Financial-toxicity screening and coverage support	Present study: uninsured status and low income were associated with higher CSS mortality. Prior literature documents substantial cancer-related financial burden [47].	Time constraints; limited financial counselors; variable eligibility for assistance programs.	Embed a brief financial-burden screen in oncology intake and route positive screens to financial counseling and assistance programs.	Screening completion; assistance-referral completion; documented resolution status.	Insurance type; financial-screen result; counseling referral; treatment-delay reason; medication-access barrier.
Rural referral pathways and telehealth-supported follow-up	Present study: rural residence and lower academic-center access were associated with poorer outcomes. Expert interpretation: referral coordination and telehealth may reduce selected access barriers.	Specialist shortages; broadband gaps; transportation; licensure and reimbursement constraints.	Use hub-and-spoke urology consultation, remote PSA review, survivorship telehealth, and explicit in-person pathways for biopsy and treatment.	Referral completion; telehealth completion; failed-connection rate; patient-reported access barriers.	Referral date; urology consultation date; telehealth modality; connection failure; PSA follow-up completion.
Facility-level treatment-concordance audit and resource review	Present study: facility characteristics and treatment patterns were associated with outcomes. Expert interpretation: facility dashboards can guide QI and referral investment.	Limited QI capacity; reporting burden; small subgroup sample sizes.	Begin with internal quality reporting; stratify measures by race/ethnicity, SES, insurance, and rurality with minimum-volume safeguards.	Quarterly audit cycles; QI project completion; reduction in unexplained treatment delays.	Risk-appropriate first-course management; TTI; no-definitive-treatment rate; facility type; tumor-board review; care-escalation reason.
Clinical-trial access and representation monitoring	Prior literature: access to resource-intensive and academic care differs across patient groups. Present study: academic-center treatment differed by subgroup.	Trial availability; eligibility criteria; travel burden; mistrust; language and consent barriers.	Pair academic-center referral with transportation support, language-concordant materials, and decentralized procedures when feasible.	Screening rate; eligibility review; refusal reasons; subgroup representation reports.	Trial screening flag; eligibility status; trial offered; enrollment status; decline reason by subgroup.

Abbreviations: EHR = electronic health record; SDOH = social determinants of health; SES = socioeconomic status; TTI = time to treatment initiation; QI = quality improvement. Recommendations distinguish present-study associations, prior literature, and expert implementation interpretation and do not include unsupported projected effect sizes.

## Data Availability

The datasets analyzed in this study are available from the National Cancer Database and SEER–Medicare subject to their respective data-use agreements. Because of licensing and privacy restrictions, the data are not publicly available from the authors.

## Abbreviations

ADT, androgen deprivation therapy; AI, artificial intelligence; API, Asian/Pacific Islander; APCCC, Advanced Prostate Cancer Consensus Conference; AUA, American Urological Association; CCI, Charlson Comorbidity Index; CFIR, Consolidated Framework for Implementation Research; CI, confidence interval; CSS, prostate cancer-specific survival; EBRT, external beam radiation therapy; EHR, electronic health record; FPL, federal poverty level; FQHC, Federally Qualified Health Center; HR, hazard ratio; ML, machine learning; NCDB, National Cancer Database; NHB, non-Hispanic Black; NHW, non-Hispanic White; PSA, prostate-specific antigen; QI, quality improvement; RE-AIM, Reach, Effectiveness, Adoption, Implementation, and Maintenance; SDOH, social determinants of health; SEER, Surveillance, Epidemiology, and End Results; SES, socioeconomic status; TTI, time to treatment initiation.
